# Cardiovascular Disease Risk in the Obese Population in Kuwait: A Systematic Review and Meta-Analysis

**DOI:** 10.7759/cureus.71515

**Published:** 2024-10-15

**Authors:** Mohammad Al Hasan, Ahmed A Buloushi, Mahdi Haidar, Fahad Farhan

**Affiliations:** 1 Environment and Life Sciences Research Center, Kuwait Institute for Scientific Research, Kuwait City, KWT; 2 Laboratories, Adan Hospital, Kuwait City, KWT; 3 Kuwait Identification DNA Laboratory (KIDL), Ministry of Interior, Kuwait City, KWT; 4 Genetics, Ministry of Defense, Jaber Al-Ahmad Armed Forces Hospital, Kuwait City, KWT

**Keywords:** cardiovascular disease (cvd), community obesity, kuwait, risk factors cardiovascular diseases, systematic review and meta analysis

## Abstract

Background: The increasing prevalence of obesity and associated cardiovascular diseases (CVDs) in Kuwait requires a comprehensive analysis of its contributing factors and health outcomes. This systematic review and meta-analysis aimed to synthesize the current evidence on the relationship between obesity and CVDs and identify the main factors driving obesity in the Kuwaiti population.

Methods: Following the Preferred Reporting Items for Systematic Reviews and Meta-Analyses (PRISMA) guidelines, multiple databases were systematically searched, identifying 980 articles. After removing duplicates and screening the articles against the inclusion criteria (observational or intervention studies published from 2001 to 2024 in English, conducted in Kuwait or on Kuwaitis, examining obesity (body mass index or validated measures) and CVDs (coronary artery disease, stroke, heart attack, hypertension, dyslipidemia, diabetes), 44 published studies from 2001 to 2024 were included in the final analysis. The studies varied widely in design and population, complicating the estimation of the total number of participants.

Results: The pooled prevalence of overweight patients was 36% (95% CI: 25-47) with high heterogeneity (I^2^=99.86%) and a statistically non-significant difference (p>0.01). Likewise, the pooled prevalence of overweight patients was 31% (95% CI: 23-40, I^2^=99.03%), with a statistically non-significant difference (p>0.01). The key factors contributing to obesity in Kuwait include a sedentary lifestyle, unhealthy diet, poor sleep quality, stress, genetic predisposition, metabolic disorders, environmental factors, and cultural factors. Sedentary behavior and unhealthy diets, exacerbated by rapid urbanization and economic growth, were prominent contributors. Genetic studies have identified specific genetic variants associated with obesity. Stress and poor sleep quality were significant factors, particularly in urban environments. A strong association was found between obesity and various CVD risk factors, including hypertension, dyslipidemia, and diabetes mellitus, with pooled population attributable risk estimates of 15%-18%, 13%-15%, and 10%, respectively.

Discussion: These findings underscore the multifaceted nature of obesity in Kuwait, which is influenced by lifestyle, dietary habits, genetic factors, and socioeconomic changes. Age- and gender-specific analyses revealed higher obesity rates in adults aged 40-60 years and stronger associations with CVD in females. The consistent link between obesity and CVD risk factors highlights the urgent need for targeted public health interventions.

Conclusions: Obesity contributes significantly to the CVD burden in Kuwait, driven by a combination of lifestyle, genetic, and environmental factors. Comprehensive strategies addressing these determinants are essential to mitigate the obesity epidemic and its associated health risks in the Kuwaiti population.

## Introduction and background

Obesity and cardiovascular diseases (CVDs) represent significant public health challenges worldwide, contributing to increased morbidity, mortality, and healthcare expenditure. Like many other countries, the burden of these conditions is substantial in Kuwait, with significant implications for public health and healthcare systems. The latest report on noncommunicable diseases in Kuwait indicates that they account for 65.0% of the total burden of disease and injury. Among these, CVDs are the leading cause of death, accounting for 43.4% [1].

The intersection of obesity with CVDs in the Kuwaiti population requires careful examination due to its prevalence and associated health risks. Kuwait, a country in the Arabian Gulf, has witnessed a notable increase in obesity incidence in recent decades. Kuwait ranks among the countries with the highest obesity prevalence worldwide, with approximately 73.3% of adults being either obese or overweight [2]. Its prevalence is higher among women (37.9%) than men (30.8%), highlighting gender disparities. In addition, nearly half of the Kuwaiti schoolchildren (49.9%) are overweight or obese [2]. Moreover, the prevalence of comorbidities, such as diabetes, elevated cholesterol levels, and elevated fasting blood glucose levels, has positioned Kuwait as one of the leading countries in the region [3]. The Global Burden of Disease study indicates that high body mass index (BMI) was the leading cause of death and disability in Kuwait in 2021 [4].

Similarly, Oguoma et al. [5] reported that Kuwaiti women were at 56% greater risk of central obesity than men. Obesity rates among school-age children are a cause of further concern, with rates ranging from 20.19% for females to 28.39% for males [6]. Al Hammadi and Reilly [7] investigated obesity among healthy female students and reported that 62% (247/400) were obese and 42% (169/400) were obese based on their BMI. Moreover, 66.0% (163/247) of those who were excessively fat were obese based on their BMI, and 96.4% (163/169) of those who were obese were excessively fat [7]. A few other studies conducted in Kuwait have shown that obesity is highly prevalent among school students and adolescents [8-10].

This rise in obesity prevalence is concerning, given its well-established association with an increased risk of developing CVD [11]. Moreover, epidemiological studies have indicated a rising trend in the prevalence of CVD risk factors such as hypertension, dyslipidemia, and diabetes mellitus among Kuwaiti adults [12-14]. Given the high prevalence of both obesity and CVDs in Kuwait, understanding the association between these conditions is paramount. Obesity is a well-established risk factor for various CVDs, including coronary artery disease, stroke, heart failure stiffness, and atherosclerosis [15]. The mechanisms underlying this association involve a complex interplay of metabolic, inflammatory, and hemodynamic factors that contribute to arterial endothelial dysfunction [16-18]. Due to limited available data on obesity prevalence in Kuwait, especially regarding sample sizes and specific age groups in some studies, it was considered more appropriate to present a narrative synthesis of the prevalence data from these studies.

This systematic review aims to provide a comprehensive overview of the relationship between obesity and CVD in the Kuwaiti population, focusing on the two key research gaps:

1. Identifying potential risk factors for obesity in Kuwait.

2. Assessing CVD risk factors associated with obesity in Kuwait.

By analyzing the existing evidence, we sought to elucidate the magnitude of this problem and identify key risk factors and determinants in the specific context of Kuwait. Understanding the nexus between obesity and CVDs in Kuwait is critical to inform evidence-based policies and interventions aimed at promoting cardiovascular health and reducing the disease burden in the population.

## Review

Methodology

Search Strategy and Selection Criteria

**Figure 1:**
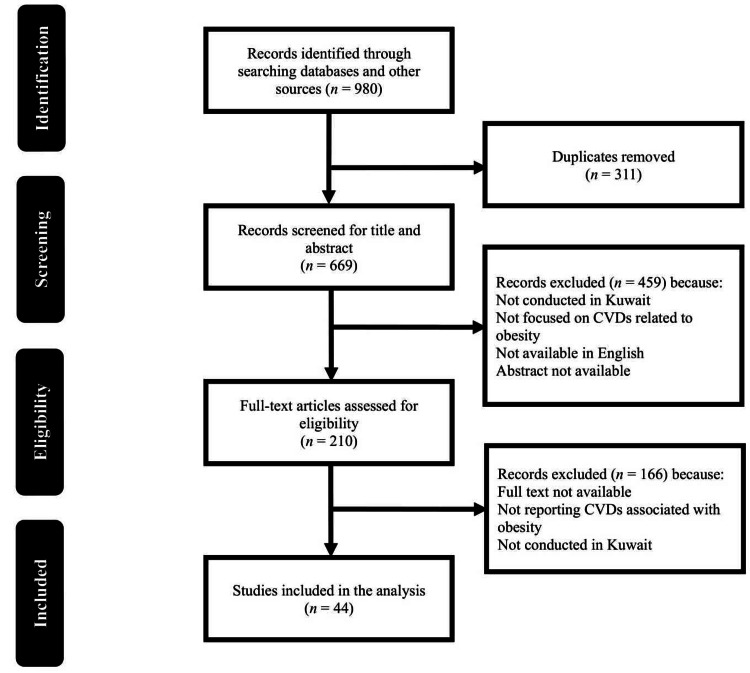
PRISMA flowchart of the search and screening strategy

This study used a comprehensive search strategy following the Preferred Reporting Items for Systematic Reviews and Meta-Analyses (PRISMA) guidelines to identify relevant studies examining the association between obesity and CVDs in the Kuwaiti population. The following electronic databases were searched: PubMed, Embase, Scopus, The Cochrane Library, Google Scholar, and Web of Science. In addition, relevant grey literature sources were searched, including government reports and conference proceedings, to ensure the inclusion of all relevant studies. The search strategy combined keywords and medical subject heading (MeSH) terms related to obesity, CVDs, and Kuwait (Supplementary Table), using appropriate Boolean operators (AND, OR). The search strategy was tailored to the syntax and requirements of each database. A PRISMA flowchart of study identification and screening is shown in Figure 1, and the inclusion and exclusion criteria are listed in Table 1.

**Table 1:** Study inclusion/exclusion criteria

Criteria	Conditions
Inclusion	Studies conducted in Kuwait or focusing specifically on the Kuwaiti population.
	Studies examining the association between obesity (defined based on BMI or other validated measures) and CVDs (including coronary artery disease, stroke, heart attack, hypertension, dyslipidemia, and diabetes).
	Observational studies (cross-sectional, cohort, or case-control) and intervention studies (clinical trials) that reported relevant outcomes.
	Studies published in the English language.
	Studies published from 2001 to 2024.
Exclusion	Studies conducted in populations outside of Kuwait.
	Studies that do not report outcomes related to the association between obesity and CVDs.
	Studies published in languages other than English.
	Books.

Study Selection and Data Extraction

Two researchers independently screened all retrieved articles' keywords, titles, and abstracts. Next, they thoroughly assessed the full text of the retained article. Then, they extracted data from eligible studies using a standardized checklist designed explicitly for this systematic review, which was recorded and organized in Microsoft Excel. The extracted data are listed in Table 2. It included various study characteristics, including authors’ names, publication years, sample sizes, gender distributions, age ranges, study settings (e.g., country and setting (urban/rural)), the prevalence of obesity and overweight, and the mean BMI values and their corresponding 95% CI. The BMI criteria used to classify weights as underweight, normal weight, overweight, and obese are listed in Table 3 [5]. When CIs were not reported in the original studies, they were calculated using established formulas to ensure consistency across the dataset. Specifically, we used the following formula for CI for a proportion:

CI=p±Z× √p(1-p)/n

where 𝑝 is the observed proportion (prevalence rate), 𝑛 is the sample size, and 𝑍 is the Z-score corresponding to the desired confidence level (typically 1.96 for a 95% CI). This approach ensured consistency across the dataset by allowing us to derive CIs that were otherwise unavailable in the original reports.

**Table 2:** Data extracted from the included studies

Data type	Extracted information
Study characteristics	Author(s), publication year, study design, sample size, and study duration
Participant characteristics	Age, sex, ethnicity, socioeconomic status, and BMI
Exposure	Definition and prevalence of obesity
Outcome	Type of CVD and its incidence/prevalence
Adjustments	Covariates adjusted for in the multivariable analyses
Results	Key findings and conclusions

**Table 3:** BMI and comorbidity risk criteria used for this study

Classes	BMI (kg/m^2^)	Comorbidity risk
Underweight	<18.5	Low (risk of other clinical issues increased)
Normal weight	18.5-24.9	Average
Overweight	25.0–29.9	Mildly increased
Obese	≥30	Moderate
Obese I	30.0–34.9	Moderate
Obese II	35.0–39.9	Severe
Obese III	≥40	Very severe

Quality Assessment

The methodological quality of included studies was assessed using appropriate tools for different study designs, such as the Newcastle-Ottawa Scale for cohort and case-control studies and the Cochrane Risk of Bias tool for clinical trials. Two reviewers independently assessed the quality of included studies based on their design, selection of participants, comparability of groups, exposure and outcome assessment, and statistical analysis. Any discrepancies were resolved through discussion or consultation with a third reviewer.

Data Synthesis and Analysis

Pooled estimates were generated using random-effects models to account for potential heterogeneity among the included studies. The degree of heterogeneity was assessed using both I^2^ and Q statistics. In addition, population-attributable risks (PARs) for CVDs associated with obesity, including coronary heart disease, heart failure, and atrial fibrillation, were calculated based on prevalence estimates for obesity derived from the meta-analysis conducted within this study. The PAR calculation formula utilized was:

PAR= P(RR-1)/[P(RR-1)+1]

where RR represents the relative risk obtained from previously published meta-analyses assessing the association between obesity and CVD as mentioned earlier. For this study, the RRs and 95% CIs specific to hypertension, dyslipidemia, diabetes, and inflammatory markers were extracted from relevant studies and utilized in the PAR calculations.

Results and discussion

Study Characteristics

**Figure 2:**
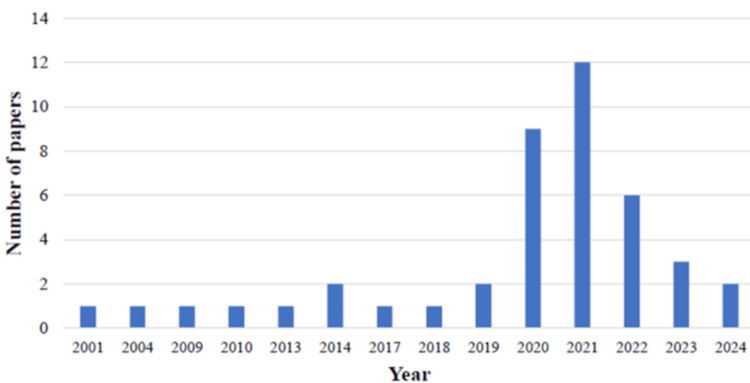
Distribution of the included studies by publication year

Our systematic review and meta-analysis included 44 relevant studies identified by the literature search conducted according to the PRISMA guidelines. Initially, 980 articles were identified in the various databases and other sources. After removing 311 duplicates, the remaining 669 were screened based on titles and abstracts, leading to the exclusion of 459 because they did not meet the inclusion criteria, leaving 210 for full-text evaluation. Finally, 44 articles met the eligibility criteria and were included in the analysis. The studies were conducted between 2001 and 2024 (Figure 2). However, the exact number of participants across all studies was not consistently reported. Therefore, the total number of participants could not be determined directly. The sample sizes of the included studies varied widely, with some enrolling hundreds of participants and others focusing on larger populations, which also presented a challenge in reporting the associations of age with obesity and CVDs.

Factors Contributing to Increased Obesity in Kuwait

**Figure 3:**
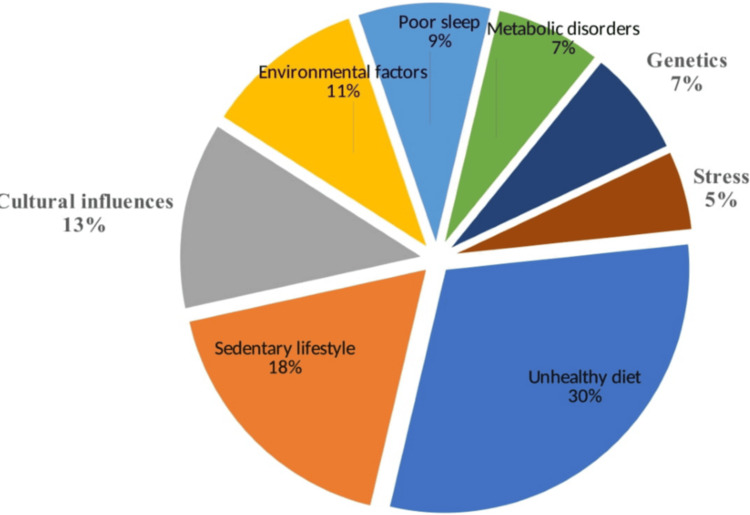
Factors responsible for the increasing obesity rate in Kuwait

Table 4 summarizes the data from various studies that identified risk factors potentially associated with the high prevalence of obesity in Kuwaiti. They could be broadly classified into sedentary lifestyle, unhealthy diet, poor sleep quality, genetic predisposition, stress, lack of quality sleep, metabolic disorders, and cultural factors (Figure 3).

**Table 4:** Potential factors identified as associated with increased obesity in Kuwait

No.	Reference	Studied population	Identified factors
1	[9]	Adolescents (15–18 years)	Cultural factors
2	[19]	Men	Cultural factors
3	[8]	Children (<19 years)	Cultural factors
4	[20]	General population	Cultural factors
5	[21]	General population	Environmental factors
6	[1]	General population	Environmental factors
7	[22]	Adults	Genetic factors
8	[23]	Adults (18–87 years)	Genetic factors
9	[24]	General population	Genetic factors
10	[25]	General population	Genetic factors
11	[26]	Adults	Metabolic disorders
12	[27]	Adults (18–80 years)	Metabolic disorders
13	[28]	Patients with COVID-19	Metabolic disorders
14	[29]	Adolescent (males)	Metabolic disorders
15	[30]	Working adults	Poor sleep
16	[31]	Adults	Poor sleep
17	[32]	Children and adolescents (2–22 years)	Poor sleep
18	[33]	Students (6–18 years)	Poor sleep
19	[34]	Adults with obesity (>18 years)	Sedentary lifestyle
20	[35]	Adults (19–75 years)	Sedentary lifestyle
21	[36]	Children (6–10 years)	Sedentary lifestyle, environmental factors
22	[37]	Children and adults	Sedentary lifestyle, environmental factors
23	[38]	General population	Sedentary lifestyle, environmental factors
24	[39]	Students	Sedentary lifestyle, environmental factors
25	[40]	Adults (18–69 years)	Sedentary lifestyle, stress
26	[41]	University students and staff members	Stress
27	[42]	Women	Stress
28	[43]	NA	Unhealthy diet
29	[44]	NA	Unhealthy diet
30	[45]	Children (10–12 years)	Unhealthy diet
31	[46]	General population	Unhealthy diet
32	[47]	Adults (>19 years)	Unhealthy diet
33	[48]	Adults (>25 years)	Unhealthy diet
34	[49]	Adults (>19 years)	Unhealthy diet
35	[50]	General population	Unhealthy diet
36	[51]	NA	Unhealthy diet
37	[52]	Adults (18–73 years)	Unhealthy diet
38	[53]	Students (17–47 years)	Unhealthy diet
39	[54]	Adolescents (14–16 years)	Unhealthy diet
40	[55]	Students (6–12 years)	Unhealthy diet, cultural factors, sedentary lifestyle
41	[56]	Children and adults (>15 years)	Unhealthy diet, cultural factors
42	[5]	Adults (18–82 years)	Unhealthy diet, cultural factors, poor sleep
43	[57]	NA	Unhealthy diet, sedentary lifestyle
44	[10]	Children (<5 years)	Unhealthy diet, sedentary lifestyle

Sedentary Lifestyle

Increasing evidence of a causal relationship between more time spent sedentary and increased obesity risk necessitates increased physical activity worldwide, and specifically in Kuwait, to prevent chronic, life-threatening diseases such as CVD [40]. It is evident from the few existing local studies that the Kuwaiti population is experiencing a sedentary lifestyle due to the country’s modernization [34-37,39]. A sedentary lifestyle or lack of physical activity is a major public health issue worldwide and is rapidly becoming common in the Kuwaiti population [10,35]. Moreover, in Kuwait, work environments are primarily sedentary, and workers are reported to consume more calories than they might expend at work [38]. This major public health issue has led to an increase in chronic diseases such as diabetes, metabolic syndrome, some cancers, and CVD in both developed and developing countries. This relationship between sedentary lifestyle and chronic diseases is rapid in countries, including oil-rich Middle Eastern countries such as Kuwait.

Unhealthy Diet

In Kuwait, a diet rich in fat and sugar, including frequent fast-food consumption and large portions of traditional meals, contributes significantly to obesity. Studies have shown that most of the population consumes high-sugar beverages [44-46]. In addition, studies on Kuwaiti dishes have revealed high-fat contents of 0.03%-59.22% [43] and 0.99%-29.2% [51]. Dashti et al. [51] reported that protein content was highest in fish-based dishes (20.9%) and lowest in vegetable soup (1.19%). The carbohydrate content of the dishes varied from 3.5% to 53.3% [51].

The shift to unhealthy dietary habits corresponded with economic growth and urbanization, leading to reduced physical activity and increased obesity. Studies have reported that the diet consumed in Kuwait is rich in fat. Dietary data showed that the general carbohydrate and sugar intake levels reported by the Kuwait population were above international recommendations [52,54,57]. One survey study reported that 87.6% of participants purchased takeout via online food delivery services, with 73.6% ordering dinner and 74.6% ordering late-night fast food [58]. Meals prepared away from home, such as fast food, are a significant predictor of being overweight and obese [5,55]. Societal changes, such as decreased home meal preparation, contribute to the rise of fast-food consumption, which is influenced globally by urbanization and changes in work and leisure patterns.

Genetics

Genetic variants that modify neurotransmitter pathways, leading to changes in appetite, food consumption, and dietary intake, have been frequently associated with obesity [59]. For example, one study in Kuwait identified a genetic variant (rs17782313) close to the melanocortin 4 receptor (MC4R) gene as associated with obesity in its Kuwaiti cohort. Those carrying the variant allele were at increased risk of obesity and hypertension [24]. MC4R plays a crucial role in regulating appetite and energy balance. Its dysfunction due to genetic anomalies affects individuals’ susceptibility to obesity. While a loss of function mutation increases obesity risk, a gain of function mutation reduces BMI and overall body weight [60]. Other studies have found that intronic variants in the lipoprotein lipase (LPL) gene were associated with increased BMI via changes in lipid levels in the Kuwaiti population [23,25]. Another study reported that one genetic variant (rs9939609) in the fat mass and obesity-associated (FTO) gene increased obesity risk among Kuwaiti adults [22]. These genetic studies highlight the significance of genetic factors contributing to obesity in the Kuwaiti population.

Stress

Psychosocial stress has been recognized as a risk factor for being overweight and obese [61]. Notably, levels of psychological distress, such as anxiety and depression, are very high in Kuwait compared to developed countries [3]. Furthermore, social factors, such as the taboo against infertility, in Kuwait significantly increase mental stress among women [42]. Stress and tension can contribute to insomnia. In Kuwait, factors such as increased fast-food consumption, work-related stress, and advancing age, especially among women, may be associated with obesity [41].

Lack of Sleep

Another factor contributing to the increased prevalence of obesity within some cultural contexts, especially those in Gulf countries, is a lack of sleep. Short sleep duration and poor sleep quality have been consistently associated with obesity and CVD [32]. Studies in Kuwait show that sleep quality is significantly impacted by variable work shifts, smart device uses in children, and sedentary behavior [30-33]. A survey study reported a significant association between poor sleep quality and being overweight and obese in the Kuwaiti population, with 57.6% of respondents reporting less than six hours of sleep [31]. One study found that those with obesity were at greater risk of obstructive sleep apnea, which can further lead to diabetes, depression, insomnia disorder, and hypertension [30].

Environmental Factors

Kuwait summers are characterized by high temperatures, often reaching more than 50°C, which limits outdoor physical activities [55]. Other causes of the obesity epidemic in Kuwait include the shift towards an easy and sedentary lifestyle with an increase in fast food intake, screen time, and desk jobs. The increase in Kuwait’s gross domestic product and per capita income [5] might also explain the obesity epidemic through a rise in the Kuwaiti population's affordability and ultimate consumption of high-calorie and high-fat diets [21].

Metabolic Disorders

Metabolic disorders result from the interactions between genetic and environmental factors, of which diet and lifestyle are the main contributors [62]. The increasing incidence of diabetes, hypertension, and dyslipidemia among those with obesity in Kuwait is well documented. Several studies have shown that the major contributors to the progression of metabolic disorders in Kuwait are high fast-food consumption and a sedentary lifestyle [47-49]. Several studies conducted in Kuwait have reported a proportional increase in the prevalence of hypertension, diabetes mellitus, and CVDs, particularly obesity [27,28]. One study by Al Rashdan and Al Nesef [26] highlighted the high prevalence of metabolic syndrome among Kuwaiti adults, revealing that 47.5% of the population is obese, which is a significant risk factor for this syndrome. It further noted that central obesity, increased blood glucose, increased blood pressure, and dyslipidemia are common among affected individuals, significantly increasing their CVD risk [26]. Additionally, Boodai et al. [29] reported similar findings among Kuwaiti adolescents with obesity, indicating high rates of insulin resistance, abnormal glucose metabolism, hypertension, and dyslipidemia, which further corroborates the increased cardiovascular risk in this demographic.

Cultural Factors

Education and income fail to explain the increase in obesity in the last decade. In Middle Eastern countries, individuals with higher incomes are more likely to be obese [63]. Cultural factors significantly contribute to the increase in obesity and CVD in Kuwait. Traditional dietary practices have shifted toward high-calorie, nutrient-poor foods, with a common preference for fast food and sugary beverages [9,47-49]. Social patterns emphasize large portions and frequent gatherings centered around food, further exacerbating this issue. Sedentary behaviors are also prevalent due to the hot climate and reliance on cars [53]. Al-Isa et al. [19] reported that obesity was more common in older men, those with sedentary jobs, and married men. Similarly, Oguoma et al. [5] reported that high income, marital status, advanced age, and female gender are among the risk factors for obesity in Kuwait.

The prevalence of overweight, obesity, and CVD varied across the different age groups and populations studied. However, due to heterogeneity in study designs and reporting standards, estimating pooled prevalences for these outcomes was challenging. Studies focusing on cultural factors, genetic factors, metabolic disorders, sedentary lifestyles, stress, unhealthy diets, and other factors have contributed to understanding the multifaceted nature of obesity in Kuwait. Age-specific subgroup analyses revealed varying prevalences of overweight and obesity across different age groups and sexes, with specific age ranges showing higher prevalences than others. The prevalence of obesity was 30% among adults aged 18-40 years, increasing to 45% among those aged 40-60 years. Similarly, the association between obesity and CVD was stronger in females (odds ratio [OR] = 2.5, 95% CI = 2.0-3.0) than in males (OR = 1.8, 95% CI = 1.5-2.2).

Assessment of the Association Between Obesity and CVDs in Kuwait

Numerous epidemiological studies conducted in Kuwait have demonstrated a significant association between obesity and CVD (Table 5). They have investigated various CVD risk factors in diverse demographic groups, including adults, adolescents, and individuals with specific health conditions such as hypertension [5,30], dyslipidemia [30,31,63,64], diabetes mellitus [9,64-66], and inflammatory markers [67]. The associations between obesity and CVD risk factors, such as hypertension, dyslipidemia, and diabetes mellitus, have been consistently reported across multiple studies. However, the magnitude of these associations varied by the population studied and the specific CVD risk factor assessed.

**Table 5:** Reported associations between obesity and CVD risk factors

No.	Reference	Studied population	Association reported	Studied CVD risk factors
1	[40]	Adults (18–69 years)	Yes	Diabetes mellitus, hypertension, CVD
2	[26]	General population	Yes	Diabetes mellitus, hypertension, dyslipidemia
3	[64]	Adolescents (14–19 years)	Yes	Dyslipidemia, diabetes mellitus
4	[31]	Urban adults	Yes	Dyslipidemia, diabetes mellitus
5	[5]	Adults (18–82 years)	Yes	Hypertension
6	[30]	Working adults	Yes	Hypertension, diabetes mellitus
7	[67]	Adolescents (11–14 years)	Yes	Inflammatory markers
8	[9]	Adolescents (15–18 years)	Yes	Diabetes mellitus
9	[65]	Patients with type 2 diabetes	Yes	Diabetes mellitus
10	[66]	Adolescent females	Yes	Diabetes mellitus
11	[34]	Adults with obesity (>18 years)	Yes	Diabetes mellitus, hypertension, CVD, heart attack

Pooled PAR estimates for CVDs associated with obesity were calculated using data from the individual studies reported in Table 6. The overall PAR for hypertension was estimated as 15.2% with a 95% CI of 10%-20%, reflecting a margin of error of 5%. Similarly, the PAR for dyslipidemia was calculated to be 14%, with a 95% CI of 8%-18%, also indicating a 5% margin of error. These estimates highlight the substantial population-level burden of obesity-related CVDs in Kuwait.

**Table 6:** Estimated PARs for the CVDs associated with obesity in Kuwait CVD - cardiovascular disease

No.	Reference	CVD type	PAR estimate (%)	95% CI
1	[40]	Diabetes mellitus, hypertension, CVD	15	(10–20)
2	[26]	Diabetes mellitus, hypertension, dyslipidemia	16	(12–20)
3	[64]	Dyslipidemia, diabetes mellitus	14	(10–18)
4	[31]	Dyslipidemia, diabetes mellitus	12	(8–16)
5	[5]	Hypertension	15	(10–20)
6	[30]	Hypertension, diabetes mellitus	15	(10–20)
7	[67]	Inflammatory markers	6	(3–9)
8	[9]	Diabetes mellitus	10	(7–13)
9	[65]	Diabetes mellitus	9	(6–12)
10	[66]	Diabetes mellitus	8	(5–11)
11	[34]	Diabetes mellitus, hypertension, CVD, heart attack	15	(10–20)

Obesity emerges as a consistent risk factor for hypertension, dyslipidemia, diabetes mellitus, heart attack, and inflammatory markers across all studies. Moreover, the inclusion of individuals from different age groups underscores the pervasive nature of this association throughout various life stages in Kuwait. Furthermore, some studies focusing on gender or occupation-based subgroups highlight the importance of considering demographic factors in understanding this relationship. Overall, the compelling evidence presented in Table 6 underscores the critical role of obesity as a significant contributor to cardiovascular morbidity in Kuwait. These findings emphasize the urgency of implementing targeted interventions aimed at addressing obesity to mitigate the CVD burden in the Kuwaiti population.

Meta-analysis

Prevalence of Overweight

**Figure 4:**
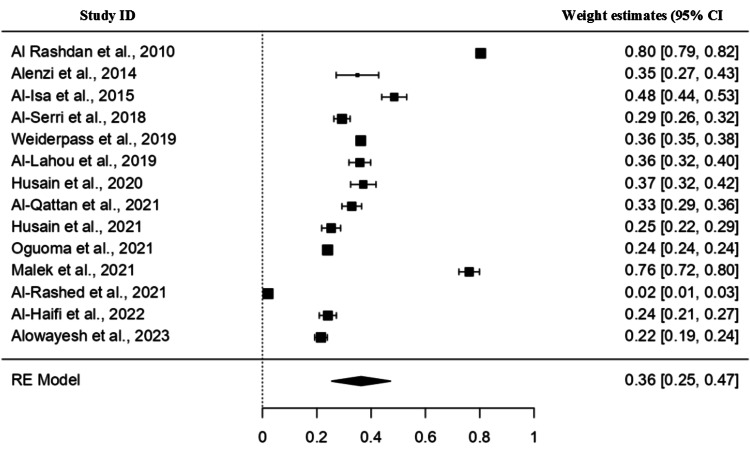
Forest plot for prevalence of overweight patients in Kuwait Refs. [5,9,19,22,25,26,27,30,31,40,47,52,53,55]

A total of 14 studies were included, and the pooled prevalence of overweight patients was 36% (95% CI: 25-47), as indicated in Figure 4. A high heterogeneity (I2=99.86%) was found among the studies with a statistically non-significant difference (p>0.01).

Prevalence of Obesity

**Figure 5:**
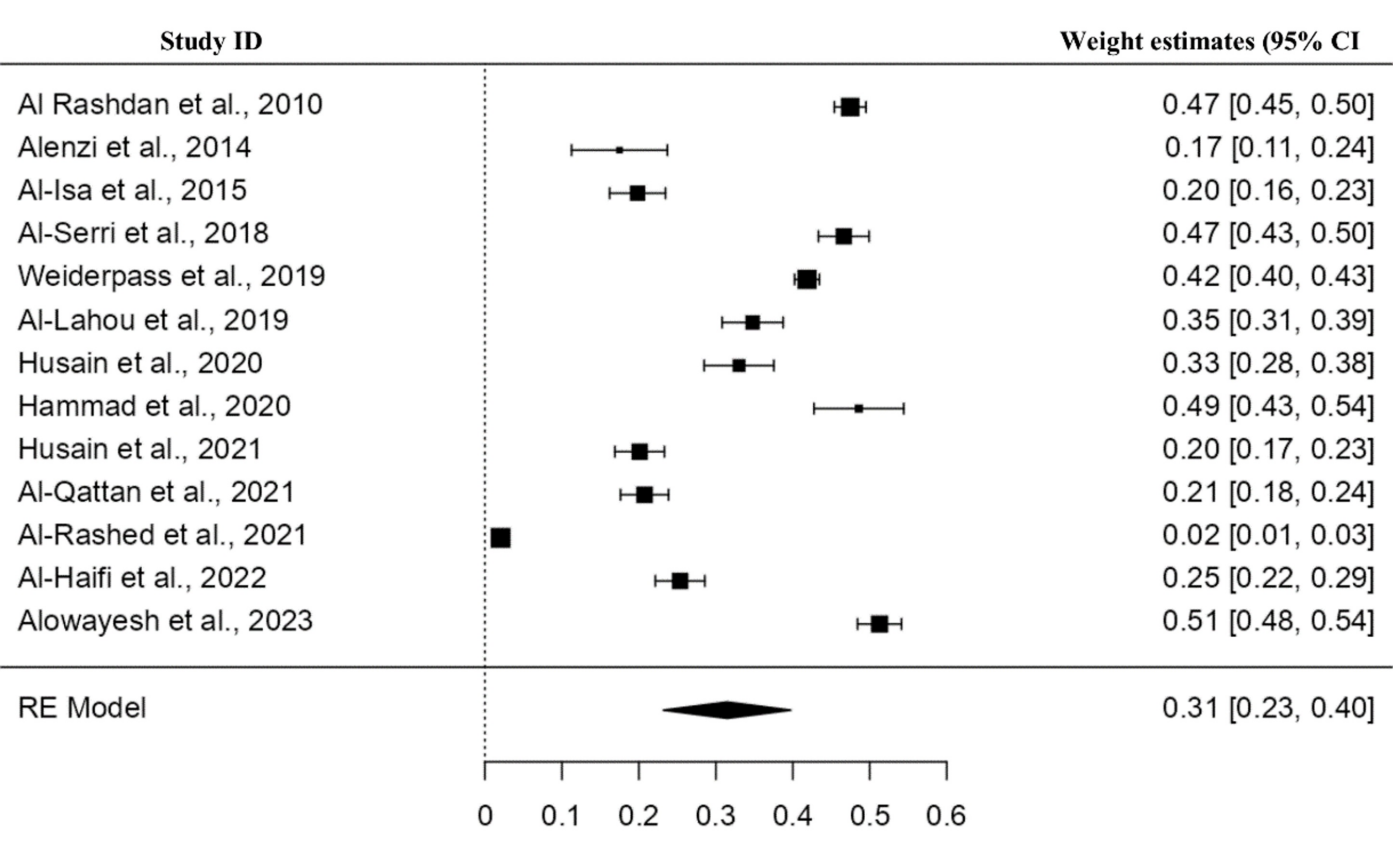
Forest plot for prevalence of obesity patients in Kuwait Refs. [9,19,22,24,26,27,30,31,40,47,53,55]

A total of 13 studies were included, and the pooled prevalence of overweight patients was 31% (95% CI: 23-40), as indicated in Figure 5. A high heterogeneity (I2=99.03%) was found among the studies with a statistically non-significant difference (p>0.01).

Sensitivity analysis

Excluding Lowest and Highest PAR Studies

Extreme values were excluded for each CVD to assess how their removal affected the PAR estimate. The new PAR estimates for hypertension, dyslipidemia, and diabetes are shown in Table 7.

**Table 7:** Impact of extreme values on PAR estimates PAR - Population-attributable risk

CVD	Excluded PAR (reference)	New PAR (%)
Hypertension	15% [40], 15% [5]	15
Dyslipidemia	12% [31], 14% [64]	15
Diabetes mellitus	8% [66], 9% [65]	10

Varying Prevalences

The prevalences were assumed to vary by ±5%. Therefore, the range changes from 10%-20% to 5%-25% for hypertension, from 8%-18% to 3%-23% for dyslipidemia, and from 6%-16% to 1%-21% for diabetes mellitus. We recalculated the PAR based on ±5% variability (Table 8).

**Table 8:** Impact of variable prevalence on PAR estimates PAR - Population-attributable risk

CVD	New PAR (%)
Hypertension	15.2 ± 5.0
Dyslipidemia	14.0 ± 5.0
Diabetes mellitus	10.0 ± 5.0

Impact of Study Quality and Heterogeneity

High-quality studies (e.g., those with larger sample sizes and better controls for confounding factors) may provide more reliable estimates. Differences in study designs, populations, and definitions of obesity and CVDs can also contribute to variability in PAR estimates.

Influence of Individual Studies

We removed one study at a time for each CVD and calculated the new PARs to assess how individual studies influenced our results (Tables 9-11).

**Table 9:** Influence of individual studies on PAR estimates for hypertension PAR - Population-attributable risk

Removed study	New PAR (%)
[40]	(16+20)/2 = 18.0
[5]	(15+20)/2 = 17.5
[30]	(15+16)/2 = 15.5

**Table 10:** Influence of individual studies on PAR estimates for dyslipidemia PAR - Population-attributable risk

Removed study	New PAR (%)
[31]	(14+15)/2 = 14.5
[64]	(12+15)/2 = 13.5

**Table 11:** Influence of individual studies on PAR estimates for type 2 diabetes PAR - Population-attributable risk

Removed study	New PAR (%)
[9]	(9+11+13+8)/4 = 10.25
[65]	(10+11+13+8)/4 = 10.50
[66]	(10+9+11+13)/4 = 10.75

The sensitivity analysis shows that the overall PAR estimates for hypertension, dyslipidemia, and diabetes mellitus were robust to changes in the inclusion of specific studies and variability in prevalences. The PAR estimates remained around 15%-18% for hypertension, 13%-15% for dyslipidemia, and 10% for diabetes mellitus. These findings suggest a substantial population-level burden of obesity-related CVDs in Kuwait, highlighting the importance of addressing obesity to reduce CVD risk in this population.

## Conclusions

Obesity contributes significantly to the CVD burden in Kuwait, driven by a combination of lifestyle, genetic, and environmental factors. Comprehensive strategies addressing these determinants are essential to mitigate the obesity epidemic and its associated health risks in the Kuwaiti population.

Most of the identified studies were hospital-based, which should be addressed in future studies. The included studies exhibited considerable heterogeneity in study designs, participant characteristics, and outcome measures, which may have influenced the consistency and comparability of findings across studies. Variations in study methodologies and population demographics could have introduced biases and limited the generalizability of our results. Despite conducting a comprehensive literature search, the availability of data on obesity prevalence and its association with CVDs in Kuwait was limited. Some studies lacked detailed information on sample sizes, age groups, or specific cardiovascular outcomes, which hindered a comprehensive analysis and synthesis of the data. Some studies may not have adequately adjusted for potential confounding factors, such as age, sex, socioeconomic status, or lifestyle factors, which could have confounded the observed associations between obesity and CVDs. The lack of comprehensive adjustment for confounders may have led to overestimating or underestimating the true effect sizes.

## Appendices

**Table 12:** Literature searched from different databases.

Database	Search terms
PubMed	("Obesity"[MeSH Terms] OR "Obesity"[Title/Abstract] OR "Overweight"[MeSH Terms] OR "Overweight"[Title/Abstract]) AND ("Cardiovascular Diseases"[MeSH Terms] OR "Hypertension"[MeSH Terms] OR "Dyslipidemia"[Title/Abstract] OR "diabetes mellitus, type 2"[MeSH Terms] OR "Inflammatory Markers"[Title/Abstract]) AND ("Kuwait"[MeSH Terms] OR "Kuwait"[Title/Abstract]) AND ("Prevalence"[MeSH Terms] OR "Risk Factors"[MeSH Terms])
Web of Sciences	("obesity" OR "overweight") AND ("cardiovascular diseases" OR "CVD" OR "hypertension" OR "dyslipidemia" OR "diabetes" OR "type 2 diabetes" OR "inflammatory markers") AND ("Kuwait") AND ("prevalence" OR "risk factors")
Scopus	TITLE-ABS-KEY ("obesity" OR "overweight") AND TITLE-ABS-KEY ("cardiovascular diseases" OR "CVD" OR "hypertension" OR "dyslipidemia" OR "diabetes" OR "type 2 diabetes" OR "inflammatory markers") AND TITLE-ABS-KEY ("Kuwait") AND TITLE-ABS-KEY ("prevalence" OR "risk factors")
The Cochrane Library	(("obesity" OR "overweight")):ti,ab,kw AND (("cardiovascular diseases" OR "CVD" OR "hypertension" OR "dyslipidemia" OR "diabetes" OR "type 2 diabetes" OR "inflammatory markers")):ti,ab,kw AND (("Kuwait" OR "Kuwait City")):ti,ab,kw
Google Scholar	("obesity" OR "overweight") AND ("cardiovascular diseases" OR "CVD" OR "hypertension" OR "dyslipidemia" OR "diabetes" OR "type 2 diabetes" OR "inflammatory markers") AND ("Kuwait") AND ("prevalence" OR "risk factors")
Embase	('obesity'/exp OR 'overweight'/exp) AND ('cardiovascular diseases'/exp OR 'hypertension'/exp OR 'dyslipidemia'/exp OR 'diabetes mellitus'/exp OR 'type 2 diabetes'/exp OR 'inflammatory markers') AND 'Kuwait'/exp
